# LncRNAs and CircRNAs in cancer

**DOI:** 10.1002/mco2.141

**Published:** 2022-05-12

**Authors:** Xin Yin, Huiran Lin, Lei Lin, Lei Miao, Jing He, Zhenjian Zhuo

**Affiliations:** ^1^ Department of Pediatric Surgery, Guangzhou Institute of Pediatrics, Guangdong Provincial Key Laboratory of Research in Structural Birth Defect Disease, Guangzhou Women and Children's Medical Center Guangzhou Medical University Guangzhou Guangdong China; ^2^ College of Pharmacy Jinan University Guangzhou Guangdong China; ^3^ Faculty of Medicine Macau University of Science and Technology Macau China; ^4^ Laboratory Animal Center, School of Chemical Biology and Biotechnology Peking University Shenzhen Graduate School Shenzhen China

**Keywords:** cancer, circRNAs, lncRNAs, neuroblastoma

## Abstract

It is well known that noncoding RNAs (ncRNAs) cannot encode proteins, but they can play important regulatory roles in tumors by combining with proteins, RNAs, and DNAs. As more and more studies reveal the important roles and underlying mechanisms of long noncoding RNAs (lncRNAs) and circular RNAs (circRNAs) in cancer, their huge application potential in cancer therapy cannot be ignored. For example, lncRNAs can be involved in tumor‐related signal transduction pathways, cell cycle control, DNA damage, epigenetic regulation, and microRNA control. A group of studies confirmed that abnormal expression of lncRNAs can affect cancer progression. Furthermore, as covalently closed continuous circular ncRNAs, many recent studies have shown that circRNAs have regulatory effects and other important biological significances in cancer. Interestingly, circRNAs were found to have translational functions. This has greatly attracted people's attention to circRNAs research. In this review, we introduce the important roles of lncRNAs and circRNAs in some representative cancers, respectively. Furthermore, we focus on the biological functions and important clinical therapeutic implications of lnRNAs and circRNAs in neuroblastoma. Our review also focuses on providing rationale and relevant references for novel biomarkers for neuroblastoma diagnosis, prognosis, and treatment.

## Graphical abstract

In this study, we comprehensively summarized the latest insights into the lncRNAs in cancer especially neuroblastoma by their functions (i.e., survival rate, proliferation, apoptosis, differentiation, cell cycle, migration, invasion, metastasis, glycolysis, DNA methylation, drug resistance, and susceptibility). The potential clinical applications of lncRNAs as prospective novel biomarkers and therapeutic targets of neuroblastoma were also discussed. In addition, we also briefly highlighted the functions of circular RNAs (circRNAs) in cancer especially neuroblastoma. This review provides a comprehensive landscape of lncRNAs and circRNAs in cancer especially neuroblastoma.

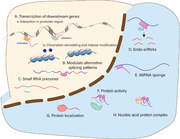

## 1 INTRODUCTION

In the past 30 years, researchers have discovered many noncoding RNAs (ncRNAs) in addition to coding RNAs.^1^ As the name suggests, these ncRNAs are not involved in coding proteins.^1^ This enriches the scientific community's understanding of RNA physiology. The ncRNAs can be divided into short RNAs and long noncoding RNAs (lncRNAs) according to a length threshold of 200.^2^ Although lncRNAs cannot encode proteins like mRNAs, they can participate in tumor progression through other mechanisms like regulating mRNAs.^3^ So far, lncRNAs have been shown to influence cancer progression, such as epigenetic regulation,^4^ DNA damage,^5^ cell cycle,^6^ and chromosomal instability,^7^ suggesting their clinical potential for cancer. And more studies focus on discovering the mechanism of lncRNAs in cancers, such as gastric cancer,^8^, ^9^ prostate cancer,^10^ breast cancer,^11^, ^12^ lung cancer,^13^, ^14^ glioma,^15^, ^16^ and osteosarcoma.^17^, ^18^

**FIGURE 1:**
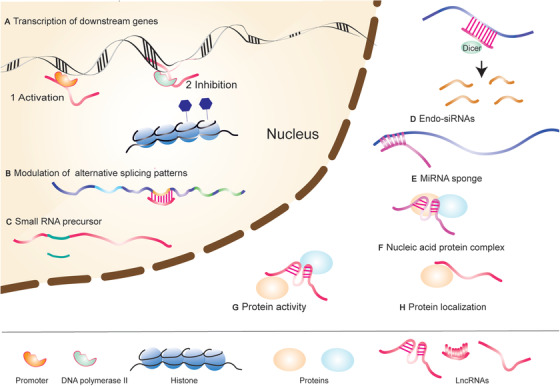
LncRNAs have multiple mechanisms in cellular processes. (A) Affecting the transcription of downstream genes, such as acting on promoter regions and affecting chromatin remodeling and histone modification; (B) regulating alternative splicing patterns; (C) acting as a small RNA precursor; (D) producing endo‐siRNA; (E) acting as a miRNA sponge; (F) forming nucleic acid‐protein complexes; (G) interacting and affects protein activity; (H) affecting protein localization (originally made on Adobe Illustrator)

LncRNAs mainly affect cancer progression by binding and interacting with RNAs, DNAs, and proteins.^19^ The regulatory role of lncRNAs in tumors is also related to their localization, sequence, and secondary structure.^20^ LncRNAs exert their regulatory roles in cells through a variety of mechanisms (Figure 1). LncRNAs can exert their important regulatory functions by acting as decoys, scaffolds, guides, and signals.^21^ As signaling molecules, lncRNAs can affect important signaling pathways by regulating the expression of downstream genes.^22^ Most lncRNAs interact with RNAs as competing endogenous RNAs (ceRNAs), thereby affecting gene expression and tumor development.^23^ These lncRNAs bind to miRNAs through their own response elements (microRNA response elements).^24^, ^25^ As a decoy, lncRNAs bind with transcriptional regulators to inhibit the corresponding pathway.^26^ Moreover, lncRNAs can also combine with regulation factors to locate the complex to specific DNA sequences.^27^ The lncRNAs have cis and trans roles here. Last, multiple translational factors can unite with a lncRNA to activate different signal pathways simultaneously. In a recent study, lncRNA SNHG9 and its associated PA (phosphatidic acids) interact with the LATS1 C‐terminal domain to promote LATS1 liquid–liquid phase separation (LLPS) and inhibit YAP phosphorylation mediated by LATS1. It is a novel regulatory role of lncRNA in cancer, facilitating the LLPS of a signaling kinase.^28^ Understanding possible commonalities of underlying mechanisms could facilitate the construction of instructive and predictive models of lncRNA function.

CircRNAs are covalently closed continuous circular ncRNAs that are usually specifically expressed in tissues.^29^ CircRNAs are very stable compared with mRNAs because they are resistant to most RNases that act on mRNAs. Interestingly, circRNAs accumulate with age in neural tissues in metazoans.^30^ This suggests circRNAs may be involved in age‐related brain diseases. For a long time, circRNAs were considered as functional RNAs and directly involved in various biological processes. For example, they can mediate gene transcription or transcriptional expression by molecular combination with miRNA, and they are involved in inhibiting miRNA‐mRNA binding and limiting downstream miRNA phenotypic effects.^29^ In recent years, emerging circRNAs have been found to have translational functions.^31^ CircRNA participated in some important diseases progress control, and they are also regarded as novel biomarkers in cancer development, progression, and drug resistance.^32^ CircRNAs have been reported to be associated with different types of cancers, including lung cancer,^33^ prostate cancer,^34^ and melanoma.^35^ CircRNAs also have numerous mechanisms in cancer. They generally act as miRNA sponges or ceRNAs to inhibit the binding of miRNAs to their targets.^36^ In the nucleus, circRNAs affect selective splicing of their linear homologs, competing with pre‐mRNAs.^37^ Furthermore, circRNAs can form specific circRNAs, binding alters protein location, transcription, and translation.^38^ CircRNAs can also be translated if the start and stop codons are preserved. The peptides translated by circRNAs play different roles in regulating tumor energy metabolism, epithelial–mesenchymal transition (EMT) metastasis, c‐Myc oncoprotein stability, and so on. The latest research on cancer about circRNAs has revealed that circDIDO1 could encode protein DIDO1‐529aa and regulate PRDX2 protein to inhibit gastric cancer.^39^ This research also showed that circRNAs play more than one role in cancer procession.

In recent years, the important functions of identifying new biomarkers in tumors have been continuously studied and revealed, such as the role of new molecular targets in the occurrence and development of tumor cells, the important signaling pathways involved, and their clinical significance in diagnosis and prognosis.^40^ Based on these mechanistic studies, developing drugs targeting these new biomarkers are popular research directions for cancer treatment.^40^ With the various functions and mechanisms in cancer (progression, development, induced therapy resistance, and dissemination), lncRNAs and circRNAs have high value in clinical use in cancer. In our review, we introduce the important roles of lncRNAs and circRNAs in some representative cancers, respectively. In addition, we focus on the intrinsic mechanisms, biological functions, and important clinical therapeutic implications of lncRNAs and circRNAs in neuroblastoma, considering limited review in this field as well as our intensive research in neuroblastoma. The current review will provide insights for developing new therapeutic targets and exploring diagnostic and prognostic markers for cancer especially neuroblastoma.

## 2 LNCRNAS AND CIRCRNAS IN CANCERS

### 2.1 Lung cancer

Lung cancer is the most deadly type of malignancy of all cancers, accounting for 2 million and 1.76 million deaths annually.^41^ Over the past 4 decades, the advances in therapeutics and early detection technologies have doubled the 5‐year survival rate of lung cancer patients, but the 5‐year survival rate is just 20%.^42^ Lung cancer often spreads to the brain, which is the leading cause of death.^43^ Tobacco smoking, the biggest risk factor for lung cancer, leads to 80–90% of this disease.^44^ The lower tobacco smoking rates have brought down the incidence and mortality of lung cancer.^45^ Lung cancer is classified by the World Health Organization into non‐small cell lung cancer (NSCLC) (85% of lung cancers) and small cell lung cancer (SCLC) (15% of lung cancers) according to the main histology, prognosis, and therapeutic significance.^46^, ^47^

LncRNA CBR3‐AS1 was overexpressed in NSCLC tissue compared with adjacent normal tissue. CBR3‐AS1 downregulation reduced invasion, proliferation, and migration. And it also promoted apoptosis of NSCLC cells and inhibited cell cycle progression. In experiments investigating the mechanism of CBR3‐AS1, CBR3‐AS1 was shown to act as an oncogene through the CBR3‐AS1/miR‐409‐3p/SOD1 pathway.^48^ CTD‐2245E15.3, a novel lncRNA, was recently found to promote NSCLC growth by interacting with and positively regulating anabolic enzymes PC and ACC1.^49^ Using RNA‐sequencing (RNA‐seq), Qian et al.^50^ characterized a novel oncogenic lncRNA LCAT3 (Lung Cancer Associated Transcript 3). They found that m6A‐regulated LCAT3 promotes lung cancer progression via binding with FUBP1 to activate c‐MYC.^50^ XIST, an oncogene in NSCLC, can suppress cell proliferation, migration, and invasion. Recently, three pathways for the role of XIST in NSCLC were disclosed. XIST can bind to EZH2 to affect KLF2 expression and can also bind to miR‐449a to affect Bcl‐2 expression.^51^ In addition, miR‐186‐5p can also interact with XIST.^51^ Recently, Ku et al.^52^ found that silencing LINC00240 suppressed the invasion and migration of lung cancer cells. Further experimental analysis revealed that LINC00240 acted as a sponge for miR‐7‐5p and induced the overexpression of EGFR. Their findings shed new light on the importance of functionally lncRNA, which represent a potential target for the treatment of lung cancer.

As an oncogene in NSCLC, high expression of circRNA C190 may affect lung cancer progression through constitutively active EGFR.^53^ The research also confirmed that MAPK/ERK pathway was the target of C190 based on the fact that C190 can regulate cyclin‐dependent kinase (CDK)s and RPS6, which could also be regulated by the MAPK/ERK pathway.^53^ CircPVT1 was a cancer promoter and could increase cell proliferation and invasion in NSCLC. In the upstream pathway, circPVT1 was upregulated by the transcription factor c‐Fos. By binding with miR‐125b, circPVT1 mediated the E2F2 expression to impact the migration, cell proliferation, apoptosis, and invasion.^54^ Consisted with this study, Qin et al.^55^ also discovered that circPVT1 knockdown suppressed cell proliferation and promoted apoptosis via the PCT1/miR‐497 pathway.

### 2.2 Breast cancer

Breast cancer remains one of the most common malignancies in women, with approximately 70–80% of patients in the early and nonmetastatic stages.^56^, ^57^ One in eight to one in 10 women is diagnosed with breast cancer.^56^, ^57^ Statistics showed that black women had a higher death rate than white women.^58^ No cure for patients with advanced and distant organ metastases and it is still curable when cancer is just in the breast or spread to the axillary lymph nodes.^59^ Genetic causes account for 10% of breast cancers. But the impact of other factors, such as environment or family size, should also be considered on breast cancer. The mortality of breast cancer has declined but the reason is still unknown.^58^ To accelerate this trend, expanding access to early detection, high‐quality prevention, and treatment service to women are ideal means.

As a heterogeneous disease, numerous lncRNAs and circRNAs are related to the etiology of breast cancer. LncRNA HOTAIR can promote proliferation and metastasis and lead to invasion and migration in breast cancer.^60^ As a ceRNA for miR‐20a‐5p, HOTAIR can bind miR‐20a‐5p and then further participate in regulating the expression of HMGA2.^60^ Another study disclosed another axis of HOTAIR in breast cancer, HOTAIR/miR‐129‐5p/FZD7 axis. The influence of HOTAIR knockdown can be reversed by the knockdown of miR‐129‐5p, such as cell proliferation, migration, invasion, and EMT. Furthermore, as a target of miR‐129‐5p, FZD7 reversed the effects of miR‐129‐5p.^61^ As a well‐known oncogene, H19 also played a crucial role in breast cancer. It upregulated EGFR and c‐Met and induced the activation of correlating downstream Akt and ERK.^62^ In addition, H19 can sponge miR‐340‐3p and reduce YWHAZ expression. H19 then counteracts proliferation, metastasis, invasion, and EMT through the YWHAZ/Wnt/β‐catenin signaling pathway.^63^ In syngeneic, xenograft, and transgenic models, overexpression of lncRNA MALAT1 has been found to inhibit breast cancer metastasis.^11^ In syngeneic, xenograft, and transgenic models, overexpression of lncRNA MALAT1 has been found to inhibit breast cancer metastasis. MALAT1 can lose its ability to bind the promoter of its target genes and the coactivator YAP by binding to protransfer transcription factors. These biological functions may explain the association of MALAT1 levels with the decreased ability of breast cancer to progress and metastasize. These findings suggest that MALAT1 may be a lncRNA that suppresses tumor metastasis.^11^ LncRNA XIST is also a common oncogene and upregulated in breast cancer. It can be inhibited by miR‐7 to exert a regulatory effect on the miR‐92b/Slug/ESA axis.^64^ The downstream of lncRNA XIST was discovered by the group of Zhao. They found that knockdown of XIST or overexpression of miR‐101 could suppress C/EBPα and KLF6 expression, and then promote cell proliferation and migration and regulate macrophage polarization.^65^ A novel lncRNA, Uc003xsl.1, was recently found to be expressed at high levels in triple‐negative breast cancer (TNBC), and high expression of Uc003xsl.1 was shown to be associated with poor prognosis in TNBC patients. Mechanistic studies have found that NKRF itself can regulate NFκB‐mediated IL8 transcriptional levels by binding to negative regulatory elements in IL8, an NFκB‐responsive gene. And Uc003xsl.1 can directly interact with the nuclear transcription factor NKRF (NFκB inhibitor) to eliminate the regulatory effect of NKRF. This process activates the NFκB/IL8 axis and promotes TNBC progression.^66^ Therefore, targeting Uc003xsl.1 in TNBC has promising therapeutic potential.

**FIGURE 2:**
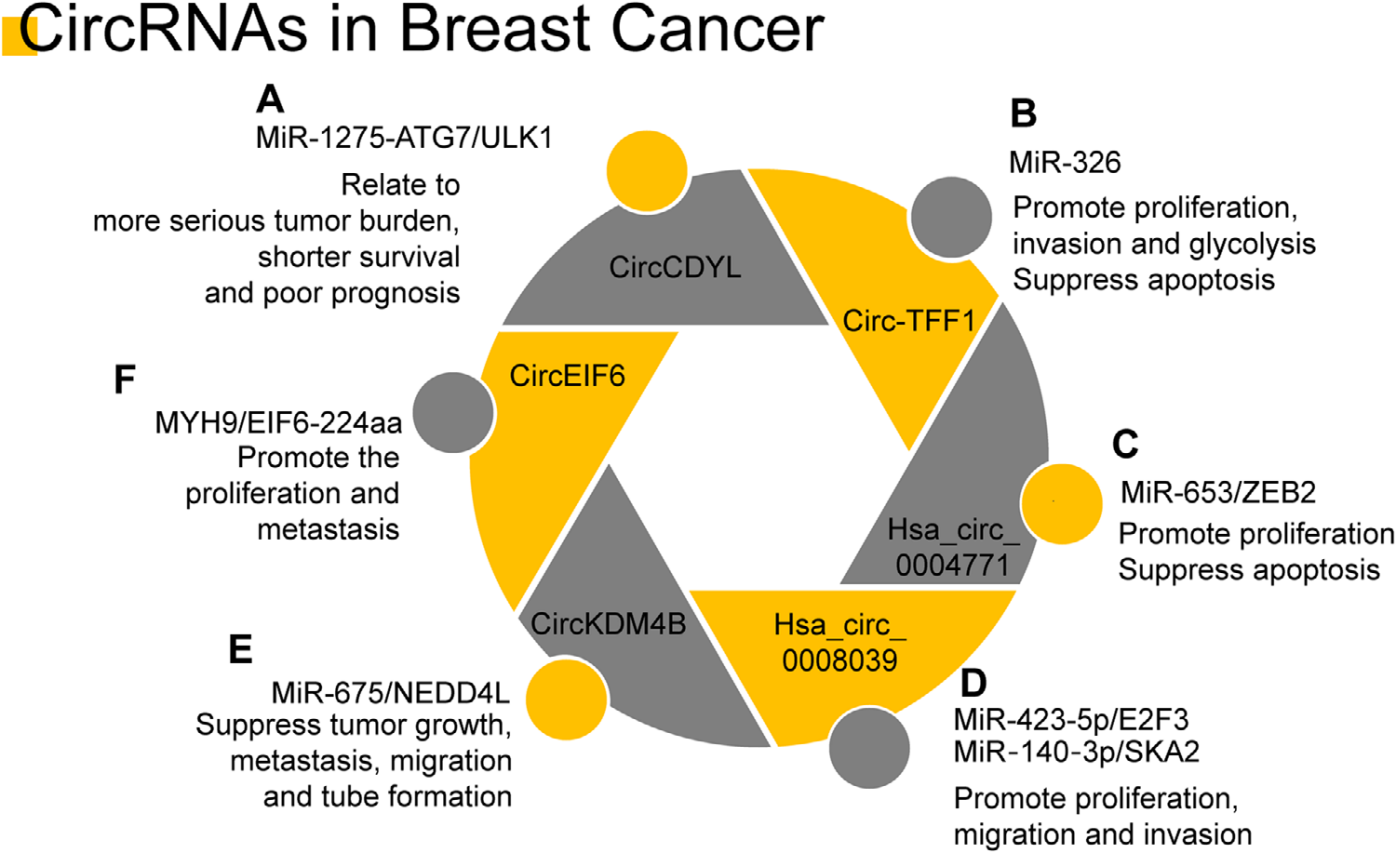
The important roles of circRNAs in breast cancer. (A) CircCDYL affects breast cancer through the miR‐1275‐ATG7/ULK1 axis. CircCDYL is associated with a greater tumor burden, shorter survival, and poor prognosis; (B) Circ‐TFF1 can be upregulated in breast cancer as a tumor promoter. It can promote cancer by interacting with miR‐338‐3p and miR‐593‐3p; (C) Hsa_circ_0004771 acts as an inducer in breast cancer. It can sponge miR‐653 and inhibit the expression of ZEB2; (D) the circRNA hsa_circ_0008039 was also upregulated in breast cancer. The hsa_circ_0008039 regulates E2F3, which is a target of miR‐423‐5p. Knockdown of hsa_circ_0008039 inhibits cell proliferation, migration, invasion, and glycolysis in breast cancer; (E) CircRNA_KDM4B suppressed tumor growth, metastasis, migration, and tube formation through the miR‐675NEDD4L axis; (F) CircEIF6 inhibit promoted the proliferation and metastasis through the MYH9/EIF6‐224aa axis (originally made on Adobe Illustrator)

CircRNAs play an important role in breast cancer (Figure 2). CircCDYL was proved to be a biomarker of poor prognostic status, and its upregulation is related to shorter survival, greater tumor burden, and poor prognosis. Mechanically, circCDYL strengthened the progression via the miR‐1275‐ATG7/ULK1 axis.^67^ Furthermore, another study confirmed the relationship between circCDYL and shorter survival.^68^ It revealed that circCDYL can sponge miR‐190a‐3p to suppress the tumor suppressor gene TP53INP1. CircTFF1 can be upregulated in breast cancer as a tumor promoter. It can sponge directly to miR‐326.^69^ Another circRNA that also upregulates in breast cancer and serves as an inducer is hsa_circ_0004771. It can sponge miR‐653 which can inhibit the expression of ZEB2.^70^ This suggests that hsa_circ_0004771 may play a role in breast cancer through hsa_circ_0004771/miR‐653/ZEB2.^70^ CircRNA hsa_circ_0008039 was upregulated in breast cancer by acting as an oncogene. Hsa_circ_0008039 knockdown also suppressed cell proliferation, migration, invasion, and glycolysis in breast cancer.^71^ Via the miR‐675/NEDD4L axis, a novel circRNA called as circKDM4B (hsa_circ_0002926) was found to inhibit breast cancer progression.^72^ More recently, a novel circRNA, circ‐EIF6, was found to have protein coding ability. Circ‐EIF6 can encode EIF6‐224aa. EIF6‐224aa can promote TNBC progression by stabilizing MYH9 and activating the Wnt/β‐catenin pathway.^73^

### 2.3 Gastric cancer

Gastric cancer is a serious health problem and the third most common cause of cancer death globally.^74^, ^75^ Gastric cancer can be anatomically divided into true gastric adenocarcinoma and gastroesophageal junction adenocarcinoma, and it can also be divided into the diffuse type and intestinal type according to the histological structure. The incidence of distal gastric cancer has been decreased, whereas the frequency of proximal gastric cancer has been increased.^76^ The number of diagnoses increased by more than 1 million each year.^75^ It accounts for 20% of the disability‐adjusted life years from cancer in males. Among all the causes of sporadic distal gastric cancer, H pylori infection is the strongest risk one,^77^ and Epstein‐Barr virus is also an important risk factor.^78^ About 1–3% of gastric cancer are hereditary.^79^ In addition to H pylori, researchers have focused more on other bacteria to explore the association between gastric microbiota and gastric cancer staging. However, the association between gastric microbiota and gastric cancer remains to be validated because the effects of diet, previous disease, or ethnic differences on patients' gastric microbiota and subsequent treatment or lifestyle changes are not considered.^80^ The influences of infection and chronic inflammation have been discovered by genetic analysis of gastric cancer, which revealed that gastric cancer was induced by infection and driven malignancy by inflammation.^81^ Individualized drug‐targeted therapy is an effective tumor treatment strategy for gastric cancer heterogeneity.^82^ LncRNA PVT1 played various roles in gastric cancer. LncRNA PVT1 has been shown to promote cell proliferation through epigenetic regulation of p15 and p16 and is associated with poor prognosis in gastric cancer.^83^ For angiogenesis in gastric cancer, PVT1 activated nuclear p‐STAT3 protein to promote VEGFA expression.^84^ In another study, PVT1 was found to regulate angiogenesis by stimulating the STAT3/VEGFA signaling axis.^84^ PVT1 may be a promising target for new gastric cancer therapy.^84^ By interacting with FOXM1, PVT1 promoted gastric cancer progression.^85^ As an oncogene, LncRNA urothelial carcinoma‐associated 1 (UCA1) was found to be often highly expressed in gastric cancer.^86^ The downstream target of UCA1 is miR‐145, which interacts with MYO6. In gastric cancer, the expression of miR‐145 was low, unlike the high expression of UCA1.^86^ This suggests that UCA1 may suppress the expression of miR‐145. The abnormal expression of UCA1 was found to suppress cell apoptosis and promote cell proliferation in gastric cancer.^86^ Interestingly, other studies have shown that UCA1 can adsorb miR‐495 and miR‐203, whereas miR‐495 can target PRL‐3 and miR‐203 can target ZEB2.^87^, ^88^ These results suggested that the communication of UCA1–miRNA–mRNA played a crucial role in gastric cancer. LncRNA EIF3J‐DT can target ATG14 and activate autophagy for chemoresistance in gastric cancer.^89^ MALAT1 plays an important part in gastric carcinogenesis. A study has disclosed that lncRNA MALAT1 affects the proliferation, invasion and migration, autophagy, chemoresistance, and poor prognosis of gastric cancer patients.^90^ Recent studies have shown that another lncRNA associated with chemoresistance is HNF1A‐AS1, which acts as a ceRNA for miR‐30b‐5p and potentiates the EIF5A2‐induced EMT process in gastric cancer.^91^ And it also enhanced the proliferation and invasion of gastric cancer cells.^92^ A previous study has shown lncRNA MACC1‐AS1 could produce antioxidants to accelerate metabolic oxygen and lower reaction oxygen species.^93^ Based on the relationship between low reactive oxygen species levels and the maintaining stemness and drug resistance,^94^, ^95^ they further confirmed the hypothesis that MACC1‐AS1 also impacts stemness and chemoresistance.^96^ HOXA11‐AS, a significantly overexpressed lncRNA in gastric cancer, is associated with poor prognosis and short survival. It scaffolds chromatin modifiers PRC2, LSD1, and DNMT1, which are involved in cell growth, invasion, migration, and apoptosis.^97^ Moreover, another research has found that HOXA11‐AS can also develop gastric cells metastasis in vivo through HOXA11‐AS/STAU1/KLF2 and HOXA11‐AS/WDR5/STAU1.^98^

CiRS‐7, the sponge for miR‐7, is a potent prognostic marker for gastric cancer. High expression of ciRS‐7 is related to poor prognosis and enhanced invasiveness of gastric cancer. Mechanically, ciRS‐7 regulated the PTEN/PI3K/AKT axis as a miRNA sponge.^99^ Acting as a modular scaffold, circMRPS35 can suppress invasion and proliferation in gastric cancer cells. It binds to the FOXO1/3a promoter regions straightly to disturb the downstream target genes (p27, p21, E‐cadherin, and Twist1).^100^ Lower expression of circCUL2 in gastric cancer tissues or cells inhibits gastric cancer and modulates cisplatin sensitivity. CircCUL2 can prevent migration, cell proliferation, and invasion by sponging up miR‐142‐3p targeting ROCK2.^101^ Different form circCUL2, circNRIP1 acted as a promoter in gastric cancer. The knockout of the circNRIP1 can restrain invasion, migration, proliferation, and the expression level of AKT1 by sponging miR‐149‐5p to further regulate the AKT1 expression. It was also found that exosomal communication could transmit circNRIP1.^102^ CircST3GAL6 has been shown to exert a tumor suppressor effect in gastric cancer through the miR‐300/FOXP2 axis. It also regulates autophagy and apoptosis, possibly through the repression of MET axis transcription by FOXP2. These results suggest that CircST3GAL6 may be a potential target for gastric cancer therapy.^103^

### 2.4 Prostate cancer

Prostate cancer impacts millions of men worldwide. Most of the tumors appear in middle to old age.^104^, ^105^ More than 70% of prostate glandular tissue is peripheral, which contributes to the primary function of the prostate in young adult men.^106^ Peripheral is also the most frequent original site of neoplasms in the aged prostate.^107^, ^108^, ^109^ The risk of prostate cancer increases with age, and most diagnosed cases are found older than 60 years of age.^110^ Therefore, countries with high life expectancy are often also at high rates of prostate cancer. The mortality of metastatic prostate cancer is predicted to more than double by 2040, even there are already more than 400,000 deaths annually caused by metastatic prostate cancer.^105^, ^111^

Based on a database of risk‐related SNPs, Guo et al.^112^ selected lncRNA PCAT1 which is related to the risk of prostate cancer to reveal the underlying mechanism. PCAT1 interacts with AR and LSD1 to promote prostate cancer progression.^112^ A previous study has shown that PCAT1 could also inhibit BRCA2 expression and modulate the stabilization of MYC as a prostate cancer promoter.^113^ As a common oncogene, MALAT‐1 also functioned in prostate cancer. Upregulation of MALAT‐1 is associated with tumor stage, high Gleason score PSA, and castration‐resistant prostate cancer, suggesting its strong therapeutic potential in prostate cancer.^114^ Moreover, Wang et al.^115^ demonstrate that Urinary MALAT‐1 is a clinically valuable biological target for predicting prostate cancer risk. MALAT1 also impacted castration‐resistant prostate cancer by interacting with EZH2 recruitment to its target loci.^116^ In prostate cancer, LncRNA UCA1 can upregulate MYO6 expression to exert oncogene activity, and it needs to “sponge” miR‐143 for upregulation.^117^ UCA1 has played a key role in studies of the effects of artesunate on prostate cancer.^118^ It is involved in the significant downregulation of artesunate and acts as a ceRNA for miR‐184.^118^

CircSMARCA5, first disclosed in prostate cancer in 2017, promotes cell proliferation and blocks apoptosis.^119^ A recent study revealed the underlying mechanism that circSMARCA5 targets miR‐423 to upregulate PDCD10 expression.^120^ On the contrary, circFoxo3 increased cell survival, migration, invasion, and chemoresistance by promoting Foxo3 expression. The target miRNA of Foxo3 in this pathway is still unknown, but the study has found that high expression of Foxo3 suppresses EMT.^121^ High expression of circ_SLC19A1 significantly promoted the growth and invasion of prostate cancer cells. Circ_SLC19A1 can sponge miR‐497 to increase the level of oncogene SEPT2, thus inducing the ERK1/2 pathway and modulating the growth and invasion of prostate cancer cells.^122^ As tumor promoters, miR‐193a‐3p and miR‐338‐3p, two miRNAs of circHIPK3 in prostate cancer, are related to the progression and development of prostate cancer. CircHIPK3 upregulated the MCL1 expression by binding with miR‐193‐3p and the overexpression of MCL1 can be abrogated by circHIPK3 knockdown.^123^ Deng et al.^124^ revealing the significant clinical potential of circ_0086722 in prostate cancer therapy, and circ_0086722 has been shown to drive prostate cancer development through the miR‐339‐5p/STAT5A axis. A recent study showed that circSOBP inhibited amoeboid migration of prostate cancer cells and inhibited invasion and migration, and miR‐141‐3p was sponged and the MYPT1/p‐MLC2 axis was regulated to participate in the tumor suppressor effect of circSOBP.^125^

### 2.5 Glioma

Glioma, a central nervous system cancer,^126^, ^127^ originates from glial cells,^128^, ^129^, ^130^, ^131^, ^132^, ^133^ with six diagnoses per 100,000 people annually.^131^, ^134^ Gliomas have recently been classified based on genetic markers, whereas histopathological features have been the predominant basis in the past.^132^, ^135^, ^136^, ^137^, ^138^ Note that isocitrate dehydrogenase 1 (IDH1) and IDH2 were used for the first layer of classification.^139^, ^140^, ^141^ The main difficulty in treatment is the diffuse infiltrative growth of gliomas and the marked intertumor and intratumor heterogeneity.^142^, ^143^ Furthermore, the etiology of gliomas is unknown.^144^ The genesis and progression of glioma are significantly related to the dysregulation of complex signaling crosstalk and multiple signaling cascades,^129^, ^145^, ^146^ whereas the malignancy correlates to neuron‐glioma circuits and neuronal activity.^147^, ^148^

Several genome‐wide association studies (GWASs) provided us with the results of the association of single nucleotide polymorphisms (SNPs) with glioma susceptibility.^149^, ^150^, ^151^, ^152^ In glioma, HOTAIR has been found related to the tumor grade, consequence, and molecular subtype. Gliomas with poor prognosis have higher HOTAIR expression, and HOTAIR expression is also higher in classical or mesenchymal subtypes.^153^ HOTAIR has numerous targets in glioma, such as PRC2 complex, miR‐326, miR‐141, miR‐125a, and miR‐148b‐3p. The inhibitory effect of EZH2 (the major PRC2 complex component) is the same as that of silencing HOTAIR.^154^ This is a potential progression mechanism for HOTAIR binding to EZH2.^154^ MiR‐326 has been found to inhibit FGF1 in gliomas by silencing HOTAIR upregulation.^155^ This reveals that the inhibitory mechanism of HOTAIR is through the HOTAIR/miR‐326/FGF1 axis.^155^ By sponging miR‐141, HOTAIR reduced the expression of SKA2, thus suppressing tumor growth in glioma.^156^ A study of the therapeutic effect of Schisandrin B on glioma found that the HOTAIR–miR‐125a–mammalian target of rapamycin (mTOR) pathway plays a key role in proliferation and invasion outcomes.^157^ Furthermore, HOTAIR also impacted the permeability of the blood tumor barrier, by binding to miR‐148b‐3p for the treatment of glioma.^158^ H19 is upregulated as an oncogene in glioma and its silencing suppresses invasion.^159^ H19 is also associated with temozolomide treatment and modulates levels of resistance genes such as MDR, MRP, and ABCG2233.^160^ LncRNA XIST (X inactivate‐specific transcript) promoted proliferation and was elevated in glioma cells. Knockdown of XIST suppresses glioma progression by elevating the expression of miR‐204‐5p.^161^ XIST also participated in the chemoresistance of glioma cells. XIST expression can prevent proliferation and enhance TMA‐induced cell inhibition. Mechanically, XIST targeted miR‐29c through transcription factor SP1 (specificity protein 1) and DNA repair protein MGMT (O6‐methylguanine‐DNA methyltransferase).^162^

CircKIF4A is overexpressed in glioma, and its knockdown prevents tumor colony formation and reduces migration and invasion by inhibiting the Wnt/β‐catenin signaling pathway and proliferation‐related signals as a ceRNA for miR‐139‐3p.^163^ Similarly, circLGMN was also upregulated in glioma, as an oncogene and related to poor prognosis. By upregulating circLGMN, the expression level of LGMN was significantly upregulated, and the underlying mechanism was that circLGMN directly adsorbed miR‐127‐3p to prevent LGMN from being degraded.^164^ Both hsa_circ_0110757 and circ_0072083 can enhance the temozolomide resistance in glioma. Differently, hsa_circ_0110757 sponged with hsa‐miR‐1298‐5p to increase the expression level of ITGA,^165^ whereas circ_0072083 bound to miR‐1252‐5p to modulate NANOG and ALKBH5.^166^

### 2.6 Hepatoblastoma

Hepatoblastoma, the most frequent malignant liver tumor in children, mainly appears in the first 2 years of life.^167^ 70% of the liver malignance origin in the hepatocyte, including hepatoblastomas and hepatocellular cancer.^168^, ^169^ The cases in males are slightly more than the cases in females. In addition, most hepatoblastomas have inherited backgrounds such as the Simpson–Golabi–Behmel syndrome, Beckwith Weidemann syndrome, familial adenomatous polyposis coli, Sotos syndrome, and constitutional trisomy.^170^ The embryonic subtype is the most common variant of hepatoblastoma, and its cells feature prominent hyperbasophilic nuclei and small cytoplasm, such as those found in the liver during the first weeks of pregnancy.^171^

LncRNA zinc finger antisense 1 (ZFAS1), as an oncogene, was highly regulated in hepatoblastoma, which is associated with poor overall survival and an aggressive phenotype. Moreover, overexpressing ZFAS1 can activate the expression of HGF/c‐Met signaling‐associated molecules, while silencing ZFAS1 has the opposite effect. Mechanistically, ZFAS1 targets the 3′‐UTR of RALY by sponging miR‐486.^172^ Knockdown of CRNDE (another lncRNA) that is also upregulated in hepatoblastoma can alleviate tumor growth and angiogenesis by modulating mTOR signaling.^173^ LncRNA TUG1 can also suppress tumor growth and angiogenesis in hepatoblastoma and is upregulated in hepatoblastoma. Its knockdown has similar effects to CRNDE and is involved in abnormal vascular proliferation in hepatoblastoma through the TUG1/miR‐34a‐5p/VEGFA axis.^174^ HOXA‐AS2 prevented HOXA3 from degradation to participate in carcinogenesis in hepatoblastoma. The regulator of HOX‐AS2 is the chromatin remodeling factor ARID1B, suggesting an important role for the ARID1B/HOXA‐AS2/HOXA3 network in hepatoblastoma.^175^ Like ZFAS1, the high expression of circHMGCS1 was also related to a poor survival rate. Furthermore, the knockdown of circHMGCS1 inhibited hepatoblastoma cells proliferation and induced apoptosis by sponging miR‐503‐5p to modulate the expression of IGF2 and IGF1R, and then it influenced the PI3K‐Akt signaling pathway.^176^ Chen et al.^177^ revealed that the highly expressed circRNA CDR1 can sponge miR‐7‐5p to affect the activity between miR‐7‐5p and KLF4. This suggests that CDR1 is an oncogene in hepatoblastoma.^177^

### 2.7 Osteosarcoma

Osteosarcoma is the most common prime malignancy of bone, especially in children and adolescents, deriving from primitive mesenchymal cells and rarely forming soft tissue. Most of the etiology of osteosarcoma is still unclear.^178^ Compared with females, males are 1.4 times more susceptible to osteosarcomas.^179^, ^180^ Although the etiology of osteosarcoma is still obscure, the risk factors for osteosarcoma have been discovered, including a history of chemotherapy or radiation, history of another benign bone lesion or Paget disease, and genetic conditions (Li‐Fraumeni syndrome, hereditary retinoblastoma, Bloom and Werner syndromes, and Rothmund‐Thomson syndrome).^181^, ^182^ The 5‐year survival rate for patients with diagnosed or recurrent metastatic osteosarcoma is less than 30%.^183^, ^184^

LncRNA KCNQ1OT1 was found to upregulate in osteosarcoma, and then it promoted cell proliferation and suppress apoptosis. It can act as a ceRNA of miR‐34c‐5p to inhibit the expression of ALDOA, thereby further participating in the Warburg effect.^185^ A novel lncRNA, ODRUL, was upregulated in osteosarcoma tissues and cell lines and correlates with poor prognosis. Further mechanism experiments revealed that ODRUL can upregulate matrix metallopeptidase (MMP)2 expression by directly interacting with miR‐3182.^18^ In osteosarcoma, SNHG10 can increase FZD3 expression by sponging miR‐182‐5p and further activate the Wnt/β‐catenin single signaling pathway in tumor cells.^186^ Most circRNAs in osteosarcoma are oncogenes. For example, circMMP9, circPVT1, and circ_001621 are associated with carcinogenesis. Similarly, all the above circRNAs act as ceRNAs, binding to miRNAs to further regulate downstream targets. CircMMP9 sponged miR‐1265 to targeted CHIL1. The knockdown of circMMP9 inhibited cell migration, proliferation, and invasion.^187^ CircCAMSAP1 promotes the progression and metastasis of osteosarcoma, and this cancer‐promoting effect may require the cavernation of miR‐145‐5p and the regulated expression of FLI1 to achieve.^188^ These indicate that circCAMSAP1 may be a potential therapeutic target for osteosarcoma treatment.^188^ Circ_001621 was discovered by circRNAs microarray and was found at very high levels in osteosarcoma. It sponged for miR‐578 to suppress the inhibition of CDK4 and MMP9.^189^

### 2.8 Wilms tumor

Wilms tumor, the most common kidney cancer of infants and young children,^190^, ^191^, ^192^ is the prototype of the junction between flummoxed organogenesis and carcinogenesis.^193^ Bilateral tumors, multifocal disease, and some genetic predisposition could lead to Wilms tumor.^194^ To be specific, Wilms tumor patients with Synchronous bilateral or multifocal tumors accounted for 10% of all cases.^195^, ^196^ However, the causes of external are still obscured, except for genetic predisposition.^197^ About 5% of people with Wilms tumor have bilateral disease.^198^ Based on the fact that the incidence rate of black Africans is the highest, whereas the Asian's is lowest, it can be deduced that genetic factors are crucial for the etiology of Wilms tumor.^199^, ^200^

**FIGURE 3:**
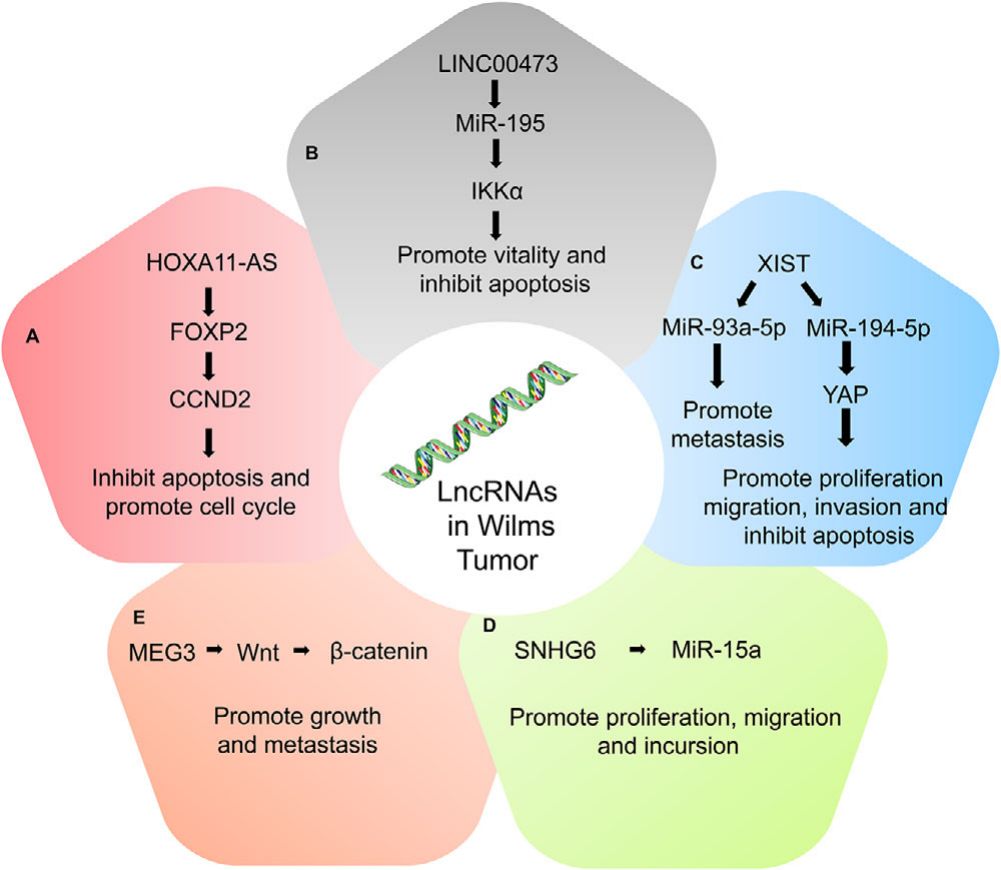
Important mechanisms and roles of lncRNAs in Wilms tumor. (A) HOXA11‐AS promotes apoptosis and maintains the cell cycle in the G1/S phase by binding to CCND2; (B) as an oncogene, LINC00473 binds to miR‐195 to promote cell viability and inhibit apoptosis; (C) the lncRNA XIST may promote migration by regulating microRNA‐193a‐5p. The lncRNA XIST can also undergo migration, invasion, and apoptosis through another axis, miR‐194‐5p/YAP; (D) SNHG6 can promote proliferation, migration and incursion through sponging miR‐15a; (E) MEG3 can promote Wilms tumor proliferation and metastasis by regulating the expression of β‐catenin through the Wnt/β‐catenin pathway (originally made on Adobe Illustrator)

The important mechanisms and roles of lncRNAs in Wilms tumor are shown in Figure 3. LncRNA XIST was significantly upregulated in Wilms tumor, which can suggest an unpleasant prognosis and the occurrence of distant metastasis^201^ and promote invasion, migration, and suppress apoptosis.^202^ Comparing the outcome of the expression of lncRNA XIST and miR‐193a‐5p, it was found that they had the opposite effect. The metastasis triggered by knockdown lncRNA XIST can be abrogated by suppressing miR‐193a‐5p. This suggests that lncRNA XIST may promote migration by regulating miR‐193a‐5p. The lncRNA XIST can also function in migration, invasion, and apoptosis through another axis, miR‐194‐5p/YAP. As an oncogene, LINC00473 binds to miR‐195 to promote cell viability and inhibit apoptosis.^203^ SNHG6 is also an oncogene that is highly expressed in Wilms tumor. Su et al.^204^ discovered that miR‐15a was the target of SNHG6, and its upregulation can inhibit the proliferation, migration, and invasion of Wilms tumor. HOXA11‐AS also plays an important role in Wilms tumor, enhancing apoptosis and maintaining the cell cycle in the G1/S phase by binding to CCND2.^205^ Unlike the aforementioned lncRNAs, MEG3 is downregulated in Wilms tumors, hindering proliferation and metastasis. Mechanistically, MEG3 can inhibit tumor cell proliferation and metastasis by regulating the expression of β‐catenin through the Wnt/β‐catenin pathway.^206^

Like MEG3, circCDYL is also downregulated in Wilms tumors, and it can further regulate Tight junction protein l through miR‐145‐5p.^207^ Similarly, circ0093740 can also enhance growth and migration in Wilms tumor and also act as a miRNA sponge. The underlying mechanism is that it sponged miR‐136 to increase the level of DNMT3A.^208^ As an oncogene, circSLC7A6 can reduce apoptosis and enhance cell viability, migration, and invasion. It was upregulated in Wilms tumor and suppressed the expression of miR‐107 to increase the level of ABL2.^209^

### 2.9 Retinoblastoma

Retinoblastoma is a malignancy that is often seen in childhood.^210^ With 8000 new cases worldwide each year, retinoblastoma accounts for 11% of childhood cancers.^211^ It starts in the womb and its progression is linked to mutated genes.^212^, ^213^ Biallelic mutations in retinoblastoma genes are major causative factors.^214^ Moreover, RB1 mutation in a nonheritable unilateral form is also a characteristic of retinoblastoma.^213^ The major cell type of development in retinoblastoma is cone precursors.^211^ Although high‐income countries have a 95% survival rate, the global survival rate is only 30% and one patient with retinoblastoma occurs for every 15,000–20,000 live births.^215^, ^216^, ^217^

**FIGURE 4:**
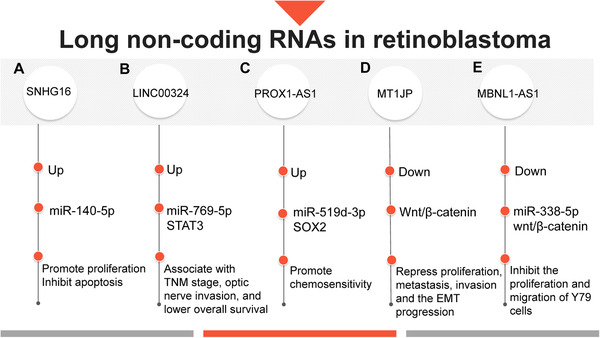
Important mechanisms and roles of lncRNAs in retinoblastoma. (A) SNHG16 is upregulated in retinoblastoma, and it promotes tumor cell proliferation and inhibits apoptosis by regulating miR‐140‐5p; (B) LINC00324 can regulate STAT3 expression by binding to miR‐769‐5p, and LINC00324 associate with TNM stage, optic nerve invasion, and lower overall survival through this pathway; (C) upregulated PROX1‐AS1 can promote chemosensitivity by regulating miR‐519d‐3p and SOX2; (D) MT1JP expression is decreased in retinoblastoma. The expression of MT1JP can repress proliferation, metastasis, invasion, and the EMT progression by inhibiting Wnt/β‐catenin pathway; (E) MBNL1‐AS1 was found to be downregulated in retinoblastoma. MBNL1‐AS1 can inhibit the proliferation and migration of Y79 cells by binding to the miR‐338‐5p and influencing Wnt/β‐catenin pathway (originally made on Adobe Illustrator)

The important roles and mechanisms of lncRNAs in retinoblastoma are shown in Figure 4. SNHG16 was abnormally upregulated in retinoblastoma. Silencing SNHG16 could repress proliferation and increase apoptosis. The influence of knockdown SNHG16 can be alleviated by silencing miR‐140‐5p, and the expression between them had a negative correlation. Taken together, the SNH16 impacted retinoblastoma by sponging miR‐140‐5p.^218^ LINC00324 is also upregulated in retinoblastoma but is associated with optic nerve invasion, TNM stage, and lower overall survival.^219^ And it promotes STAT3 expression as a ceRNA of miR‐769‐5p.^219^ LncRNA PROX1‐AS1 was upregulated in retinoblastoma tissues and cells, especially in drug‐resistant cells. In retinoblastoma, knockdown of PROX1‐AS1 promotes chemosensitivity, and this tumor suppressor effect requires PROX1‐AS1 to upregulate miR‐519d‐3p and target SOX2.^220^ In retinoblastoma, LncRNA MBNL1‐AS1 can inhibit the Wnt/β‐catenin signaling pathway to exert antitumor effect, and the pathway inhibition needs to be achieved by targeting miR‐338‐5p through MBNL1‐AS1.^221^ Similarly, the LncRNA MT1JP inhibits tumor growth by negatively regulating the Wnt/β‐catenin signaling pathway, suggesting that MT1JP may serve as a prognostic biomarker and therapeutic target.^222^

CircDHDDS is upregulated in retinoblastoma and is related to cell cycle programming, migration, proliferation, and invasion. The suppression of miR‐361‐3p can reverse the influence of knockdown circDHDDS. From the results that overexpression of miR‐361‐3p can inhibit retinoblastoma progression by binding to WNT3A, the circDHDDS/miR‐361‐3p/WNT3A axis is a potential pathway.^223^ Hsa_circ_0001649 was crucial in many cancers. For retinoblastoma, it can repress growth and enhance apoptosis by the signal pathway AKT/mTOR.^224^ In contrast, circ‐FAM158A served as an oncogene in retinoblastoma. It promoted proliferation and migration and suppressed apoptosis. Mechanically, circ‐FAM158A sponged miR‐138‐5p to regulate the expression of SLC7A5.^225^ The level of circ_0099198 and LRP6 was abnormally high, whereas the level of miR‐1287 was low in the retinoblastoma cells. Silencing circ_0099198 can inhibit proliferation and metastasis by modulating the miR‐1287/LRP6 axis.^226^

## 3 LNCRNAS AND CIRCRNAS IN NEUROBLASTOMA

Neuroblastoma is a malignant pediatric cancer in the sympathetic nervous system.^227^, ^228^, ^229^ It progresses during fetal or early postnatal life from sympathetic cells deriving from the neural crest.^230^ Neuroblastoma is characterized by heterogeneity, which can naturally regress or mature without treatment, or have a dismal outcome with potent multimodal treatment.^231^, ^232^, ^233^ However, neuroblastoma is also the most usual extracranial tumor in children, apart from obstinacy, accounting for 12% of childhood tumor‐related deaths and 7% of childhood malignancies. Although patients with non‐high‐risk neuroblastoma have an overall survival rate of more than 90%, the overall survival rate of the high‐risk patients is less than 50%, which suggests that more researches are still required to discover novel prognostic factors and therapeutic targets to develop better treatment strategies.^234^, ^235^

Recently, studying the roles of lncRNAs and circRNAs in neuroblastoma is an emerging research topic. It brings new perspectives to the prognosis and treatment of high‐risk patients.^233^ The studies of lncRNAs and circRNAs in neuroblastoma are usually performed in the following order. First, lncRNAs and circRNAs, associated with important clinical features, such as MYCN amplification or patient survival cohorts, were identified by analyzing differential expression between clinical tumors or cell lines.^236^, ^237^ Then, studies analyze the top candidates to confirm an active role in neuroblastoma cell phenotypes, such as proliferation or cell viability.^233^ Next, the potential mechanisms of action of lncRNAs and circRNAs will be understood by discovering downstream binding targets. Finally, based on these elucidated functions, the researchers explored the clinical implications of their findings and whether they could potentially translate into new diagnostic or therapeutic strategies for cancer. In this review, we focus on the significance of lncRNAs and circRNAs in neuroblastoma. Furthermore, we creatively summarize the features and commonalities between lncRNAs and circRNAs, and we also compare the mechanisms and functions of lncRNAs and circRNAs between other cancers and neuroblastoma. It provides new ideas for the diagnosis and prognostic markers of neuroblastoma and the development of new therapeutic targets.

### 3.1 LncRNAs and CircRNAs have numerous roles in neuroblastoma

**FIGURE 5:**
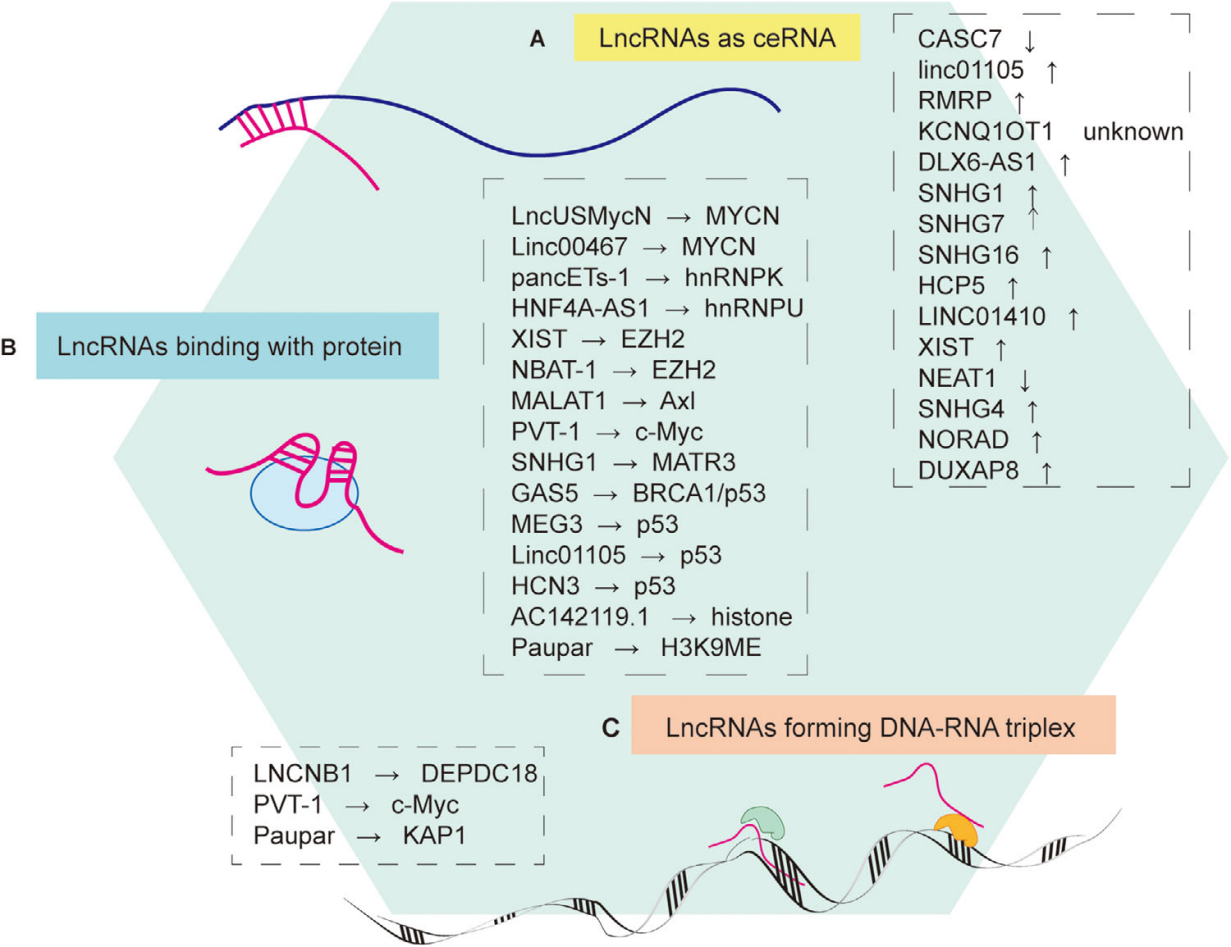
LncRNAs take action mainly in three locations: RNA, protein, DNA. (A) LncRNAs binding with mRNA primarily act as ceRNA; (B) LncRNAs bind with proteins to interact with the functions or locations of the proteins; (C) LncRNAs bind with DNAs, forming a DNA–RNA triplex (originally made on Adobe Illustrator)

Chemo‐resistance of cancer cells is influenced by the activity of lncRNAs.^238^, ^239^ A recent study has revealed lncRNA ANRIL enhanced cancer resistance by promoting homologous recombination‐mediated DNA repair.^240^ In addition, LINC00842 promoted malignancy through metabolic remodeling.^241^ LncRNA PLANE enhanced pathogenesis by regulating alternative splicing programs.^242^ LIMIT improved the immunity in cancer.^243^ These are the most recent research progress of lncRNAs in cancer. These precise characterizations in their phenotypic outputs are pivotal to discovering appropriate candidates for new clinical diagnostic and therapeutic strategies. However, the roles of lncRNAs are not comprehensively investigated in neuroblastoma. LncRNA can combine with RNA, protein, and DNA to play an important regulatory role in neuroblastoma (Figure 5). The function of lncRNAs in neuroblastoma includes their impacts on the survival rate, regulating proliferation, apoptosis, differentiation, cell cycle, migration, invasion and metastasis, glycolysis, DNA methylation, drug resistance, the risk of neuroblastoma, thus promoting or restraining the development of neuroblastoma or affecting the therapeutic outcome, acting as a tumor suppressor or oncogene (Table 1). Most researches about lncRNAs/circRNAs in neuroblastoma focus on the signal pathway or mRNA target but not the immune response. Only one study in 2021 has identified 11 immune‐related lncRNAs in neuroblastoma and the underlying mechanisms still need further study.^244^ With the growing maturity of immunization therapy in cancer, the study of immune‐related lncRNAs and circRNAs in neuroblastoma need to keep up with this trend, and we should do more research to fill in this gap.

**TABLE 1:** Representative lncRNAs in neuroblastoma

lncRNA	Expression	Functions	Related gene	Role	References
LncNB1	Up	Enhances the protein of N‐MYC	DEPDC18	Oncogene	^271^
HNF4A‐as1	Up	Promotes glycolysis	MYCN	Oncogene	^264^
DLX6‐AS1	Up	Suppresses differentiation and apoptosis, promotes proliferation, migration	STAT2	Oncogene	^265^, ^281^
MEG3	Up	Suppresses proliferation and promotes apoptosis	EMT‐related genes	Tumor suppressive	^252^
HCN3	Down	Promotes apoptosis	P53	Tumor suppressive	^252^
Linc01105	Down	Promotes apoptosis	P53	Tumor suppressive	^252^
XIST	Up	Promotes growth, migration, and invasion	DKK1	Oncogene	^276^
SNHG1	Up	Relates to the poor prognosis	miR‐338‐3p	Oncogene	^269^
CCAT2	Up	Suppresses proliferation and promotes apoptosis	P53	Tumor suppressive	^259^
DUXAP8	Up	Promotes proliferation	miR‐29	Oncogene	^260^
KCNQ1OT1	/	Promotes apoptosis	Bax	Tumor suppressive	^280^
RMRP	Up	Promotes proliferation, migration, and invasion	TACR1	Oncogene	^256^
FOXD3‐AS1	Down	Induces differentiation and decreases aggressiveness	/	Tumor suppressive	^294^
LINC01010	Down	Indicates better survival	/	Tumor suppressive	^261^
CXCR2P1	/	Exhibits critical roles in overall survival	/	/	^262^
LOC387720	/	Exhibits critical roles in overall survival	/	/	^262^
DUX4L3	/	Exhibits critical roles in overall survival	/	/	^262^
PancEts‐1	Up	Promotes growth, invasion, and metastasis	β‐Catenin	Oncogene	^253^
CASC11	Up	Promotes proliferation and invasiveness	miR‐676‐3p	Oncogene	^279^
SNHG16	Up	Promotes progression	/	Oncogene	^249^, ^254^, ^269^, ^298^
HOTAIR	Up	Risk of neuroblastoma	/	/	^296^

**FIGURE 6:**
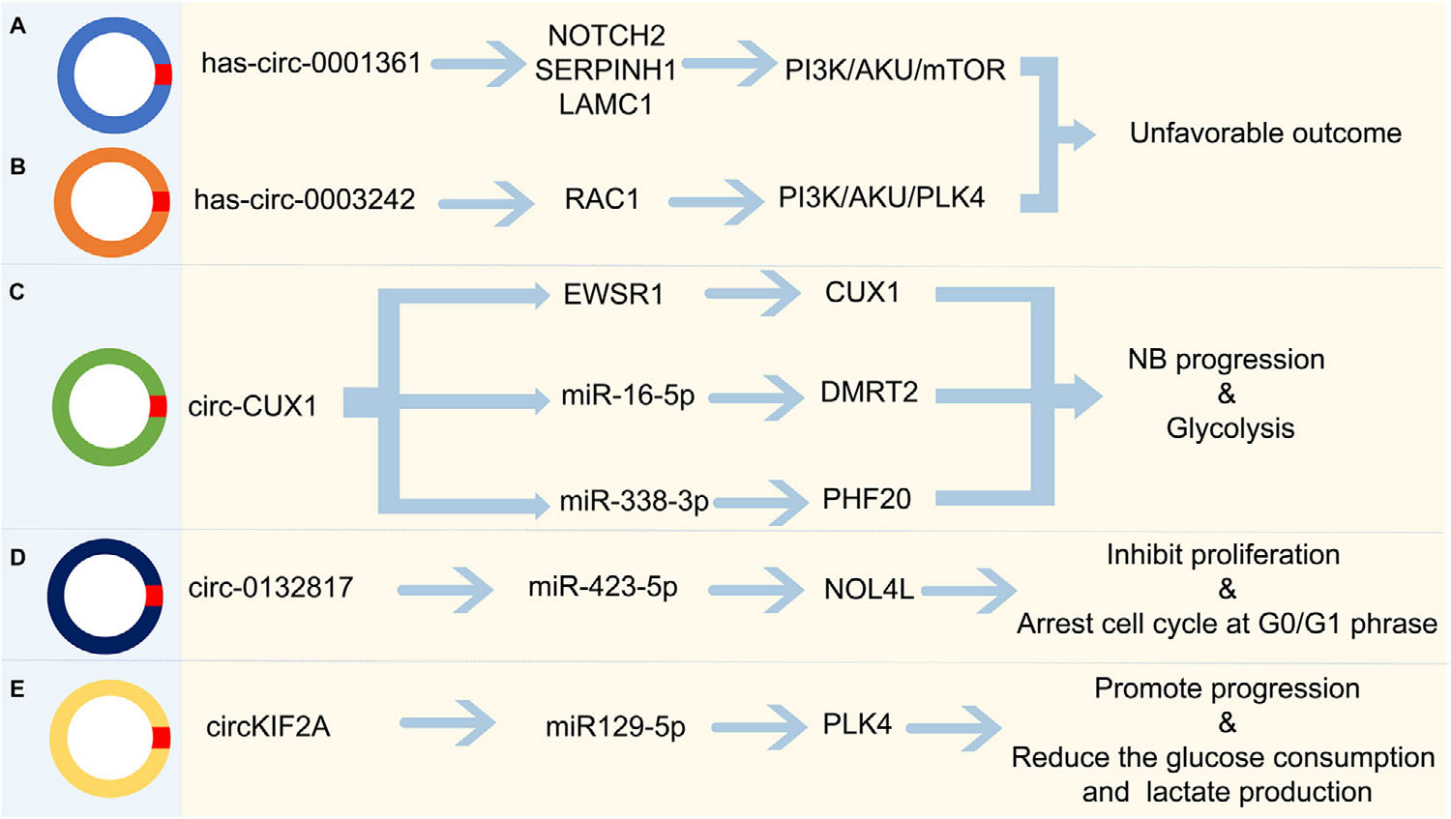
Multiple circular RNAs (circRNAs) are involved in neuroblastoma oncogenesis and progression. (A) Hsa_circ_0001361 might mediate EMT via NOTCH2, SERPINH1, and LAMC1. EMT was mediated by Polo‐like kinase 4 (PLK4) via PI3K/Akt signaling in neuroblastoma; (B) Hsa_circ_0002343 might regulate the PI3K/Akt/mTOR signaling via RAC1; (C) CircCUX1 promotes neuroblastoma progression and glycolysis by regulating the EWSR1/CUX1, miR‐16‐5P/DMRT2, miR‐338‐3p/PHF20 axis; (D) Circ_0132817 facilitates cell proliferation, migration, invasion, and glycolysis by regulating the miR‐432‐5p/NOL4L axis in neuroblastoma; (E) CircKIF2A contributes to cell proliferation, migration, invasion and glycolysis in human neuroblastoma by regulating the miR‐129‐5p/PLK4 axis (originally made on Adobe Illustrator)

Compared with lncRNAs, the roles of circRNAs in neuroblastoma were found just in the last 4 years. The regulatory mechanisms and effects of various circRNAs on the occurrence and progression of neuroblastoma are shown in Figure 6. Recent study identified more than 183,000 circRNAs by reconstructing full‐length circRNAs from long RNA‐seq reads or identifying backsplice sites from RNA‐seq reads.^245^, ^246^ Currently, RNase R and Ribo‐Zero are the gold standard methods for detecting circRNAs. In classical RNA‐seq datasets containing mainly polyadenylated RNAs, most circRNAs were not detected due to the lack of three polyadenylated tails.^247^ Some of the circRNAs have been discovered overexpressing in cancer. Moreover, circRNAs function by acting as ceRNA and interacting with RNAs.^248^

There are some overlaps between the functions of lncRNAs and circRNAs in neuroblastoma, such as affecting the migration, proliferation, invasion, and glycolysis. The functions of circRNAs have not been discovered in neuroblastoma, including mediating EMT, the production of lactate and ATP.^249^ Although the roles of circRNAs in neuroblastoma still need further exploration, the total value to the physiology and pathology of neuroblastoma is indubitability. More studies regarding the relationship between the roles of lncRNAs and circRNAs are required to accelerate the study of circRNAs in neuroblastoma. First, to find out these lncRNAs functions, we need to identify the expression pattern and binding targets of the lncRNAs through online tools—calculator program, software, chip cards, such as Coding Potential Calculator algorithm version2 (CPC2), CNCI, CPAT, ESTScan, GeneMarkS‐T.^250^ We then need to validate selected lncRNAs and discover whether differentiation of known lncRNAs caused significant changes or developmental or therapeutic effects in neuroblastoma cells. Finally, we need to further identify the biological functions of lncRNAs by downregulation or overexpression, or knockdown of them. Notably, traditional methods of studying mRNAs do not always affect lncRNAs due to their unique characteristics and low copy numbers. Sometimes it does not work when using tissue‐specific signatures to find biomarkers, they appear in the nucleus in the same way.^251^ Therefore, we need higher targeting, resolution, and operability at the molecular level.

#### 3.1.1 The examples for migration, proliferation, apoptosis, and invasion in neuroblastoma

Based on differentially expressed lncRNAs between para‐tumor tissues and tumor, Tang et al.^252^ identified 4802 lncRNAs and 5130 mRNAs that are differently expressed in neuroblastoma tissues. The three most aberrantly expressed lncRNAs were linc01105, MEG3, and HCN3. Further studies showed that linc01105 knockdown promoted cell proliferation, in contrast to the result of MEG3 overexpression. MEG3 overexpression, Linc01105 knockdown, and HCN3 knockdown all increased apoptosis. Separately, overexpression of MEG3 not only inhibits the proliferation but also promotes apoptosis of BE(2)‐C neuroblastoma cells. While knockdown of HCN3 promoted cell apoptosis and did not affect cell proliferation. However, whether the commonalities between them can interact through the same pathway or opposite actions remains to be further investigated. The recent study also found that lncRNA pancEts‐1 promoted the invasion, growth, and metastasis of neuroblastoma cells in vitro and in vivo. The knockdown of pancEts‐1 suppressed neuroblastoma progression. Using two independent short hairpin RNAs to deplete pancEts‐1, numerous viable cells were decreased significantly by BE (2)‐C and IMR32 cell lines, which reduced invasiveness by stable knockdown of pancEts‐1. In nude mice, subcutaneous injection of neuroblastoma cells firmly transfected with pancEts‐1 specific short hairpin RNAs resulted in the growth of xenografts, and tail vein injection of BE (2)‐C cells stably transfected with pancEts‐1 specific short hairpin RNAs led to fewer lung metastatic sites and grater survival probability.^253^ In one study, it has displayed that silencing SNHG16 could repress cell proliferation by inducing cell cycle arrested at the G0/G1 phase.^249^ In addition, SNHG16 silencing inhibited migration, proliferation, and invasion and induced apoptosis of neuroblastoma cells.^254^ Silencing LINC01296 not only promotes apoptosis but also inhibits neuroblastoma cell migration and proliferation. Moreover, LINC01296 knockdown was found to inhibit tumor growth in vivo.^255^ In neuroblastoma cells, RMRP knockdown can inhibit invasion and migration.^256^ Attenuated CASC15‐S, a lncRNA, increases cell growth and migration in human neuroblastoma cells.^257^ In conclusion, lncRNAs mostly have multiple roles in the progression of neuroblastoma.

#### 3.1.2 The example for poor survival rates

Improving the poor survival rates for high‐risk patients is a trend in treating neuroblastoma. However, about 40% of the high‐risk patients have poor overall survival despite comprehensive treatment.^258^ LncRNA CCAT2 expression is an independent risk factor for prognosis in children with neuroblastoma.^259^ According to the GEO database (GSE12460) and clinical data, high expression of DUXAP8 is associated with advanced neuroblastoma, and DUXAP8 expression is negatively correlated with overall survival in neuroblastoma patients.^260^ The differential expression of lncRNAs (DElncRNAs) and their risk signatures were identified by the Limma plus Voom package in R based on RNA‐sequencing data downloaded from the Genotype‐Tissue Expression database and the Therapeutical Applicable Research to Generate Effective Treatments database. DElncRNAs and one of them (LNC01010) were found to be significantly related with the survival outcome of patients with neuroblastoma in GSE62564.^261^ SVR‐NB is an overall survival time estimator and can also be used to identify lncRNA signatures that are associated with overall survival in neuroblastoma patients. Using SVR‐NB, 35 lncRNAs have been identified, showing that 4 lncRNAs (LOC729770, CXCR2P1, LOC387720, and DUX4L3) may affect the overall survival of neuroblastoma patients.^262^

#### 3.1.3 The examples for aerobic glycolysis

Unlike its anaerobic counterpart, aerobic glycolysis typically occurs in cancer and is known as the “Warburg effect.”^263^ It is one of the earliest known evidence of alternative tumor metabolism, and neuroblastoma is no exception. It is also a way that lncRNAs and circRNAs affect neuroblastoma progression. One recent study has shown that HNF4A and HNF4A‐AS1 improve tumorigenesis and invasiveness by promoting glycolytic processes, lactate production, glucose uptake, and ATP levels in neuroblastoma cells.^264^ Furthermore, P1‐HNF4A isoforms function differently in tumors due to the nature of their interactome, including their binding partners involved in gene transcription. This deserves further study. Moreover, one research on DLX6‐AS1 indicated that depletion of DLX6‐AS1 inhibited neuroblastoma cell glycolysis in vitro.^265^ CircCUX1 also contributed to aerobic glycolysis and neuroblastoma progression.^266^ High expression of circCUX1 represented a lower survival probability. CUX1 acts independently on progression and poor outcome and is a promoter of ENO1, GPI, and PGK1 expression in neuroblastoma. All of these target mRNAs played important roles in the tumor progression such as metabolic tumor promoter, an interactor of the glycolytic pathway, and ATP generation. This suggests the oncogenic roles of CUX1 in neuroblastoma. Mice that were injected with neuroblastoma cells stably expressing sh‐circCUX1 possessed smaller xenograft tumors.^267^ In the study by Yang et al. silencing of circKIF2A reduced glucose consumption and lactate production. This shows its oncogene role in neuroblastoma.^268^

#### 3.1.4 The example for drug resistance

LncRNAs and circRNAs also impact the drug resistance of neuroblastoma cells. SNHG16 silencing reduced cisplatin resistance, downregulation of, multidrug‐resistance gene 1‐type P‐gp (p‐glycoprotein) expression, MRP1 (multidrug resistance protein 1) expression and suppression of migration, proliferation, and invasion. SNHG16 silencing also reduces apoptosis during cisplatin treatment of neuroblastoma cells. SNHG16 knockdown also increased the ability of cisplatin cytotoxicity in tumor growth in vivo.^269^

### 3.2 The mechanisms of LncRNAs and CircRNAs in neuroblastoma

**FIGURE 7:**
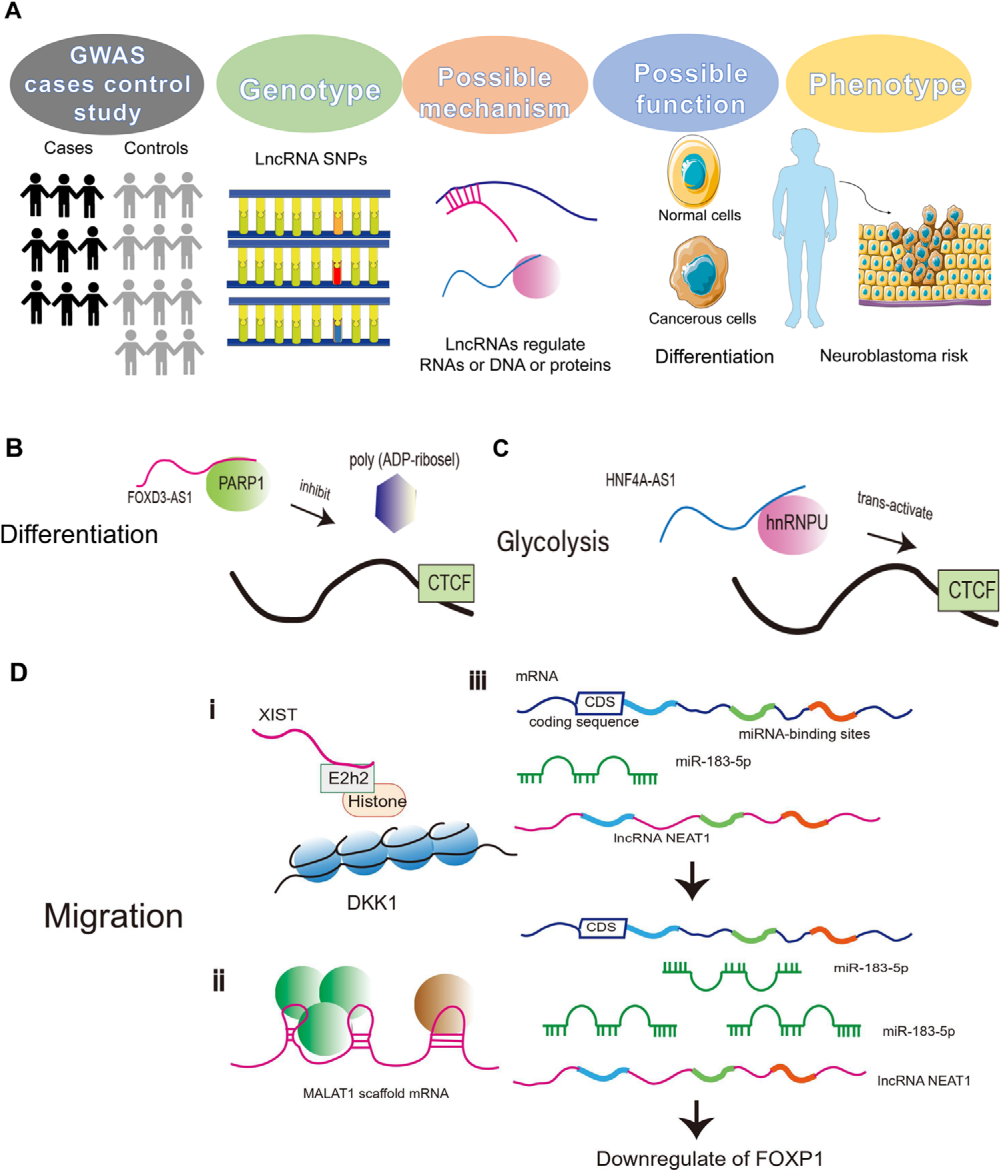
The process of identifying the association between lncRNA polymorphisms and neuroblastoma susceptibility and representative functional mechanisms of lncRNA in neuroblastoma. (A) LncRNA SNPs can confer the susceptibility of neuroblastoma. Based on the GWAS and cases control study, we can identify lncRNA SNPs related to neuroblastoma. Further analysis can disclose the possible mechanism and functions of the selected lncRNA SNPs. Finally, we can analyze the relationship between lncRNA SNPs and neuroblastoma risk according to the different phenotypes. Here are some representative functional mechanisms of lncRNA in neuroblastoma; (B) to the differentiation role of lncRNA in neuroblastoma, lncRNA, FOXD3‐AS1 interacts with poly (ADP‐ribose) polymerase 1 (PARP1) to inhibit the poly (ADP‐ribosyl) action and activate of CCCTC‐binding factor (CTCF), resulting in derepressed expression of downstream tumor‐suppressive genes; (C) to the glycolysis role of lncRNA in neuroblastoma, HNF4A‐AS1 combined with isomeric ribonucleoprotein and heterogeneous nuclear ribonucleoprotein U (hnRNPU) to trans‐activate CTCF, thus promoting the expression of glycolytic genes HK2 and SLC2A1 in neuroblastoma cells; (D) to the migration role of lncRNA in neuroblastoma (originally made on Adobe Illustrator)

To clarify the mechanism of action of lncRNAs, we first need to identify abnormally expressed lncRNAs in neuroblastoma by mining public microarray datasets. The downstream target genes are predicted by bioinformatics analysis. RIP (RNA immunoprecipitation) and luciferase reporter analysis further demonstrated the results. Due to the differences between lncRNAs and circRNAs, some things need to be paid attention to in research.^270^ LncRNAs lack conservation and circRNAs are more conserved than lncRNAs. Therefore, it is necessary to determine whether the human and mouse lncRNA sequences are identical before the experiment. The identification process of the correlation between lncRNA polymorphism and neuroblastoma susceptibility and the representative functional mechanism of lncRNA in neuroblastoma are shown in Figure 7.

#### 3.2.1 Examples of binding with protein

The binding of pancEts‐1 and hnRNPK can promote the interaction between β‐catenin and lncRNA pancEts‐1.^253^ The tumor‐promoting function of pancTts‐1 can be mediated through regulating the hnRNPK activity in part. In biotinylated pancTts‐1 pull‐down, mass spectrometry revealed that hnRNPK was the protein with the highest spectral count. Exon 2 of pancEts‐1 could interact with hnRNPK protein. It was further concluded that pancEts‐1 could stabilize protein to modulate downstream gene targets. The effects of nuclear translocation can be altered by overexpression or silencing of pancEts‐1. LncNB1 can bind to another protein called RPL35 in the cytoplasm, and its high expression is related with poor prognosis in neuroblastoma patients.^271^ It has been proved that the knockdown of lncNB1 could prevent the interaction between E2F1 protein and DEPDC18 gene core promoter. E2F1 siRNA reduced DEPDC1B mRNA and protein expression. This is identical to the effect of lncNB1 and DEPDC1B siRNA on N‐Myc protein phosphorylation, ERK phosphorylation, and expression. The mechanism was that lncNB1 in neuroblastoma cells enhanced the synthesis of E2F1 protein by binding with ribosomal protein RPL35 and directly bonding to the gene promoter of the DEPDC1B to promote the transcription of the DEPDC18 gene. Moreover, DEPDC1B induced ERK protein phosphorylation and enhanced N‐MYC protein stability. A novel intronic circRNA generated from the AGO2 gene (circAGO2) has been identified, which also interacts with proteins.^272^ It is upregulated in colon cancer,^273^ gastric cancer,^274^ neuroblastoma, and prostate cancer,^275^ and it is associated with poors patient prognosis. CircAGO2 could activate human antigen R (HuR) protein and promote the accumulation of the target gene's 3′‐untranslated region, thereby suppressing the interaction between AGO2 and its target miRNA. HNF4A‐AS1 interacts with hnRNPU protein in BE (2)‐C cells of neuroblastoma cells.^264^ HNF4A‐AS1 promoted the interaction with the CTCF (CCCTC‐binding factor) by binding with hnRNPU (heterogeneous nuclear ribonucleoprotein U) to change the transcription of HNF4A and relative genes. And then transcription factor HNF4A increased the expression of HK2 (hexokinase 2) and SLC2A1 (solute carrier family 2 member 1). HNF4A‐AS1 improves tumorigenesis, aerobic glycolysis, and invasiveness of neuroblastoma cells through the aforementioned processes. Linc01105 influences cell death pathways.^252^ HIF‐1α expression inhibits cell proliferation. And HIF‐1α expression was elevated when linc01105 is downregulated. This suggests linc01105 regulated HIF‐1α protein expression to impact proliferation. Knockdown of HCN3 increases Bid expression and promotes apoptosis, and linc01105 knockdown had the same effect. In the p53 pathway, both Bid and Noxa are apoptosis‐related proteins. Downregulation of linc01105 increased the protein levels of Noxa and Bid and enhanced apoptosis. These suggest that HCN3 and linc01105 regulate apoptosis through Noxa and Bid proteins. It has already been described above that MEG3 overexpression promoted apoptosis. However, MEG3 overexpression could not alter the levels of HIF‐1α mRNA or protein levels and reduce the expression of Noxa and Bid, which were both positively correlated with apoptosis. This indicates that MEG3 may directly promote apoptosis in neuroblastoma cells. Histone methylation is also a way lncRNAs influence neuroblastoma. For example, lncRNA XIST interacted with the histone‐modifying enzyme EZH2.^276^ The histone transacetylase inhibitor DZNep has been found to cause DDK1 protein expression, suggesting that EZH2 can inhibit DKK1 expression by inducing H3 histone methylation. XIST inhibits DKK1 expression through binding to EZH2 and H3 histone methylation. The role and relationship of DKK1 DNA methylation in neuroblastoma deserves further investigation in the future.

#### 3.2.2 Examples of interacting with miRNA/DNA

A study reported that lncRNA SNHG1 promoted tumorigenesis by increasing the level of PLK4 and sponging miR‐338‐3p in neuroblastoma. SNHG1 and PLK4 were increased in neuroblastoma tissue and cells, whereas the expression of miR‐338‐3p decreased and targets of miR‐338‐3p and SNHG1 or PLK4 were elucidated. This suggests that PLK4 directly targets miR‐338‐3p and that miR‐338‐3p can bind to SNHG1. PLK4 can inhibit the anticancer activity of miR‐338‐3p on migration and invasion.^277^ Another study showed that NR2F‐AS1 was highly expressed and miR‐493 was lowly expressed in neuroblastoma tissues and cell lines. NR2F1‐AS1 can target the downstream gene TRIM2 of miR‐493. NR2F1‐AS1 has been found to regulate TRIM2 expression by sponging miR‐493.^278^ The study determined the relationship between miR‐676‐3p, CASC11, and NOL4L based on bioinformatics tools and found that high expression of NOL4L reversed the effects of CASC11 on neonatal neuroblastoma, including inhibition of proliferation or proliferation. CASC11 upregulated NOL4L by sponging miR‐676‐3p whose expression was negatively related to CASC11.^279^ LncRNA KCNQ1OT1 can regulate miR‐296‐5p.^280^ miR‐296‐5p can be sponged by KCNQ1OT1 and reverse its effect on neuroblastoma cell apoptosis. In addition, miR‐296‐5p is directly bound to the 3′UTR of Bax mRNA. These findings indicated that KCNQ1OT1 sponged miR‐296‐5p and upregulated Bax in neuroblastoma. The expression of miR‐206 was significantly reduced and negatively correlated with the expression of RMRP in neuroblastoma cells.^256^ The miRcode online website showed some complementary sites between miR‐206 and RMRP. RMRP can interact with miR‐206 in neuroblastoma cells, such as sequestering miR‐206 from TACR1 as a ceRNA for miR‐206, implying that RMRP regulates the miR‐206/TACR1 axis to play an oncogenic role in neuroblastoma gene action. Furthermore, TACR1 overexpression induces the ERK1/2 axis and reverses the effects of RMRP downregulation on neuroblastoma cell growth. Inhibition of ERK1/2 pathway and TACR1 reduces the function of RMRP to promote proliferation in neuroblastoma cells. The knockdown of RMRP inhibits neuroblastoma xenograft growth by modulating the miR‐206 or TACR1 axis and inactivating the ERK1/2 pathway in vivo. The influence of SNHG16 overexpression reversed the effects of miR‐128‐3p‐mediated on preventing proliferation, migration, invasion, and promoting apoptosis of neuroblastoma cells.^249^ ln contrast, the elevating expression of miR‐128‐3p can be rescued by the impact of HOXA7 overexpression, suggesting that miR‐128‐3p functioned as the ceRNA of HOXA7 and miR‐128‐3p is involved in neuroblastoma progression in vitro. The lncRNA SNHG16 is the ceRNA of miR‐128‐3p. SNHG16 silencing reduced HOXA7 expression in neuroblastoma cells, and downregulation of miR‐128‐3p expression reversed this effect. These results suggest that SNHG16 directly interacts with miR‐128‐3p and dissociates miR‐128‐3p from its target gene HOXA7. DLX6‐AS1 is another lncRNA that also has a tumor‐suppressive role in neuroblastoma. It has been confirmed that miR‐107 was the downstream target of DLX6‐AS1, and miR‐107 targeted an oncogene in neuroblastoma named brain‐derived neurotrophic factor (BDNF). The suppression of neuroblastoma progression caused by DLX6‐AS1 silencing can be reversed by overexpression of BDNF or knockdown of miR‐107. In conclusion, DLX6‐AS1 promoted neuroblastoma progression by regulating miR‐107/BDNF pathway.^281^ Another research found that DLX6‐AS1 could also modulate the expression of miR‐506‐3p and STAT2.^265^ As the target of DLX6‐AS1, the knockdown of miR‐506‐3p could abrogate the reduction of DLX6‐AS1. STAT2 expression was positively correlated with DLX6‐AS1 expression in neuroblastoma tissues. And by targeting miR‐506‐3p, DLX6‐AS1 can directly regulate the expression of STAT2. The STAT family should be paid more attention to in the study of neuroblastoma.

Moreover, recent studies also discovered some circRNAs have similar functions in neuroblastoma. Circ‐CUX1 binds to the RRM region of EWSR1 and promotes MAZ transactivation, thereby altering the transcription of CUX1 and the association of other genes with neuroblastoma progression.^266^ Another group demonstrated that circ‐CUX1 accelerated the proliferation, invasion, migration, and glycolysis of neuroblastoma cells by targeting the miR‐16‐5p/DMRT2 signaling cascade.^267^ Moreover, circKIF2A could sponge miR‐129‐5p to regulate the expression of PLK4 and promote neuroblastoma progression.^268^

### 3.3 Meaningful clinical potential of LncRNAs and CircRNAs in neuroblastoma

To date, the main approaches to target lncRNAs include post‐transcriptional RNA degradation pathways, using genome‐editing techniques, modulation of lncRNA genes by steric blockade of the promoter, preventing secondary structure formation, and creating steric inhibition of RNA–protein interactions.^282^ Different functions represent various meanings for treatment. Since lncRNAs are stable in serum or plasma, they can serve as potent biomarkers for diagnosing neuroblastoma.^271^, ^283^

Given that most lncRNAs and circRNAs function as ceRNAs, direct targeting of lncRNAs or circRNAs could be more effective in neuroblastoma therapy. The RNAi pathway refers to the use of synthetic siRNA to target RNA in human cells and mouse models.^284^, ^285^ Due to the difficulty of siRNA delivery, it is still mainly used as a tool rather than a treatment, which indicates that addressing the delivery of siRNA is an important direction for future clinical research.^286^ What is more, as circRNAs have more miRNA binding sites than linear RNAs, it can be more efficient to inhibit miRNA. ASO is an ideal approach to achieve significant lncRNA knockdown for clinical trials in cancer.^287^ It can alter or inhibit gene expression through steric hindrance, splicing changes, initiation of target degradation, or other events. It works for cytoplasmic RNAs and functions effectively in the cell nucleus.^288^ There is still no therapy for ASOs in neuroblastoma. The reasons why nucleic acid‐based therapies have not been applied to the treatment of neuroblastoma mainly include the following aspects. The first puzzle is how to cross the cytoplasmic membrane. Other interacting factors, such as cellular nucleases and the innate immune response to foreign RNA, may inhibit the cellular uptake of these molecules.^289^, ^290^ Furthermore, entrapping synthetic ASOs in endosomal compartments significantly reduces the bioavailability of these molecules.^291^ Finally, we should consider oligonucleotides with minimal toxicity but unaffected therapeutic efficacy.

Based on the feasibility of transcriptional silencing of lncRNAs using CRISPR‐based methods, it may be used in the future to therapeutically target these molecules at the transcriptional level.^292^, ^293^ Therefore, it may be easier to target lncRNAs involved in blood if CRISPR‐based approaches are considered. While several preclinical studies are utilizing CRISPR‐mediated editing methods, it remains unclear whether these procedures are applicable in the clinical setting of cancer‐related diseases. Treatment with FOXD3‐AS1 constructs or siRNA targeting PARP1 or CTCF reduces tumor growth and prolongs survival in tumor‐bearing nude mice xenografts.^294^ FOXD3‐AS1 can inhibit the oncogenic effects of PARP1 or CTCF and play an important role in all‐trans retinoic acid‐mediated neuroblastoma therapy.

SNPs are among the high‐risk alterations associated with cancer occurrence.^295^ 85% of SNP are annotated in noncoding regions and related to disease development. These abnormalities could influence the expression and function of lncRNA downstream targets. Evaluating the association between lncRNA polymorphisms and neuroblastoma risk based on a large sample size and stratification are novel ways to excavate more helpful biomarker information. In the research that explored the relationships between HOTAIR gene polymorphisms and neuroblastoma susceptibility, our group genotyped six polymorphisms (rs920778 A>G, rs874945 C>T, rs12826786 C>T, rs7958904 G>C, rs4759314 A>G, and rs1899663 C>A) of the HOTAIR gene in 2 Chinese populations including 812 healthy controls and 393 neuroblastoma cases.^296^ The strength of the associations was evaluated using odds ratios and 95% confidence intervals. Three HOTAIR SNPs (rs920778 A>G, rs4759314 A>G, and rs7958904 G>C) were detected with no association between these SNPs and neuroblastoma susceptibility. Further stratification analyses found that the rs874945 C>T (*p* = 0.020), rs12826786 C>T (*p* = 0.013), and rs1899663 C > A (*p* = 0.029) polymorphisms related to neuroblastoma risk. In stratification analyses, these associations were more predominant in females and patients with tumors in the retroperitoneal region or mediastinum. This study sheds light on the association of important HOTAIR gene polymorphisms with neuroblastoma susceptibility. More GWASs are needed in the future to refine the genomics and genetics of neuroblastoma. In addition, it was found that cancer cells are more active in glycolysis than normal cells and use much more glucose to obtain more ATP for metabolic activities.^297^ Since then, new approaches and therapies targeting metabolism have become an exciting topic in cancer research. It shows that with knowledge of the key players in cancer cell glycolysis, treatments will be more accurate. Some of the lncRNAs /circRNAs exactly participate in the glycolysis, such as HNF4A,^264^ DLX6‐AS1,^265^ circCUX1,^266^ implying their important values in clinical application.

### 3.4 More LncRNAs or CircRNAs with clinical value

**FIGURE 8:**
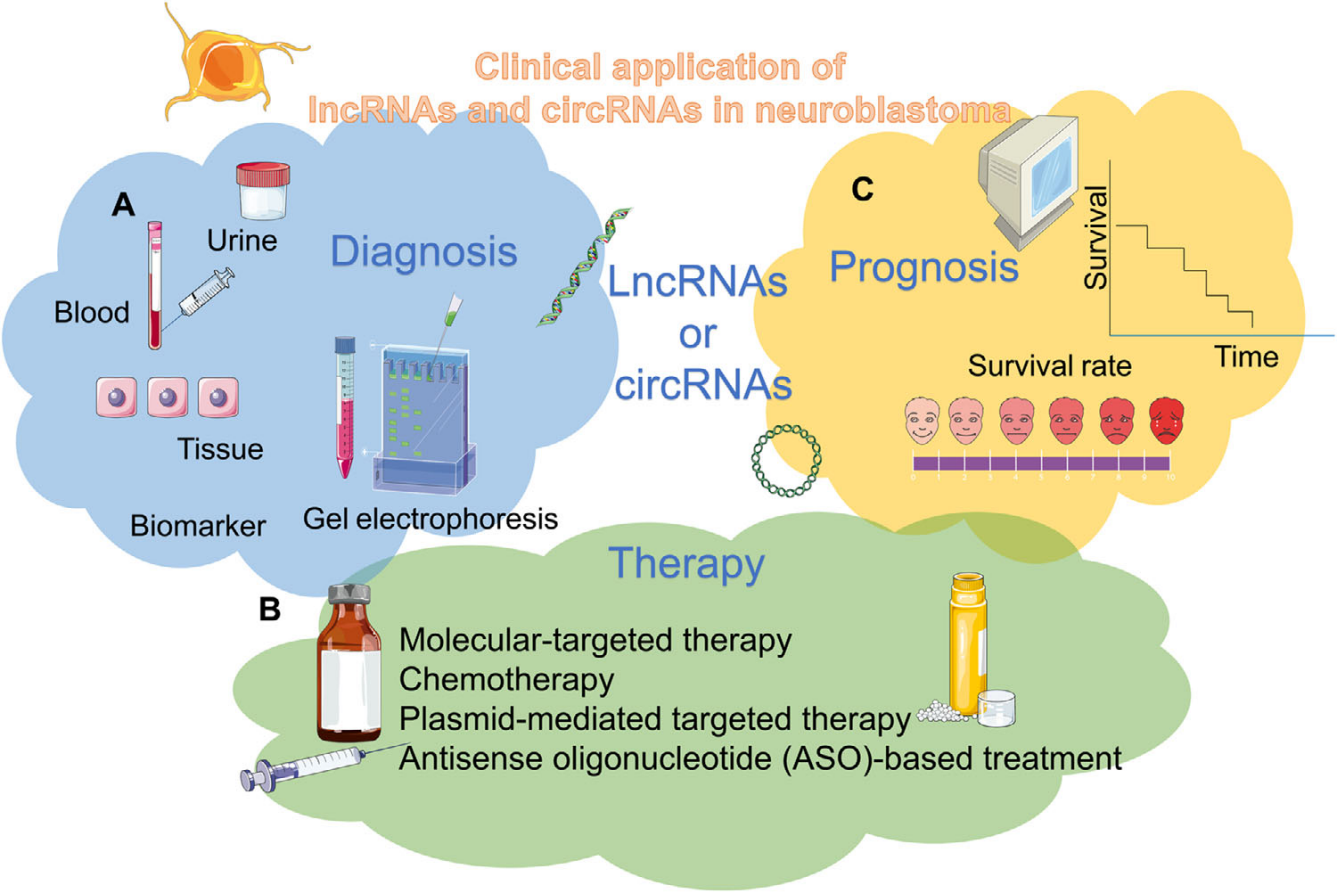
The applications of lncRNAs/circRNAs in the clinic of neuroblastoma. (A) The study of lncRNAs/circRNAs related to neuroblastoma can be used in clinical diagnosis, serving as a biomarker, and the source of lncRNAs/circRNAs can be urine and blood; (B) many therapies can also many therapies can be used based on the mechanisms of lncRNAs/circRNAs, such as molecular‐targeted therapy, chemotherapy, plasmid‐mediated targeted therapy, an antisense oligonucleotide (ASO)‐based treatment; (C) through the study of lncRNAs/circRNAs, we can also predict the survival rate based on case analysis (originally made on Adobe Illustrator)

LncRNAs/circRNAs have important clinical applications in neuroblastoma (Figure 8). Higher LINC01010 expression was significantly related to better survival, suggesting that LINC01010 is a potentially potent prognostic target in neuroblastoma.^261^ Studies have shown that HNF4A‐AS1 levels are elevated in the serum of neuroblastoma cases and are associated with the clinicopathological role of the tumor. This suggests the potential value of HNF4A‐AS1 as a biomarker for the diagnosis of neuroblastoma.^264^

Research using SVR‐NB, a method based on optimized support vector regression (SVR), has been mentioned. Sathipati et al.^262^ found four lncRNAs (LOC729770, CXCR2P1, LOC387720, and DUX4L3) could be potential biomarkers of neuroblastoma survival time. Although with the limitation of inadequate sample size, this study also showed the power of computation models in identifying lncRNA's roles in neuroblastoma. SNHG16 had essential functions in neuroblastoma therapy.^298^ GATA3 affects neuroblastoma proliferation by regulating epigenetic transcriptional control networks. It suggested that the GATA3 transcriptional network was a promising target for novel neuroblastoma therapies.^299^

SNHG16 affects the activation of the PI3K/AKT pathway in cisplatin‐resistant neuroblastoma cells, and SNHG16 does this by regulating PLK4 expression by sponging miR‐338‐3p and the SNHG16/miR‐338‐3p/PLK4 axis effect.^269^ The effect of miR‐338‐3p repression could alleviate the result of SNHG16 knockdown to cisplatin resistance. And PLK4 overexpression reversed the decrease of cisplatin resistance induced by miR‐338‐3p re‐expression. But the role of the PI3K/AKT pathway in cisplatin resistance in neuroblastoma remains unknown. Moreover, the detailed function of SNHG16 on drug resistance phenotype is still vague. The study still needs a larger disease cohort to validate these conclusions. These studies suggest that our future studies should clarify the function of the PI3K/AKT pathway in cisplatin resistance and focus on how SNHG16 modulates the resistance phenotype in both in vivo and in vitro experiments. CUX1 is independently related to poor outcomes, and patients with high circCUX1 expression had a lower survival probability in clinical neuroblastoma cases.^266^ Furthermore, administration of inhibitory peptides that block circCUX1‐EWSR1 interaction or lentiviruses that mediate circCUX1 knockdown suppressed aerobic glycolysis, invasiveness, and growth of neuroblastoma cells. This suggests the circCUX1/EWSR1/MAZ axis can be a therapeutic target for aerobic glycolysis and neuroblastoma progression. Before clinical treatment, administration of lentivirus‐mediated short hairpin RNA targeting circAGO2 inhibited downstream target genes’ expression and suppressed the tumorigenesis and aggressiveness of xenografts in nude mice.^272^ Moreover, preventing the interaction between circAGO2 and HuR repressed the carcinogenesis and aggressiveness of cancer cells. These results indicated circAGO2 helped explore novel clinical therapy for neuroblastoma. By detecting and comparing the expression of circDGKB in blood samples from neuroblastoma patients and healthy subjects, the expression level of circDGKB was significantly increased in neuroblastoma patients.^300^ Furthermore, the level of circDGKB had an essential clinical value in diagnosing neuroblastoma with an area under the curve (AUC) of 0.7778. The results of the Kaplan–Meier analysis showed that patients with a high level of circDGKB had lower survival rates. In conclusion, circDGKB might function as a diagnostic marker for neuroblastoma.

## 4 CONCLUDING REMARKS AND FUTURE PERSPECTIVES

An increasing number of studies have shown that lncRNAs and circRNAs play critical roles in tumor progression, but the understanding of lncRNAs and circRNAs in neuroblastoma is far from resolved. The involvement of lncRNAs and circRNAs in cancer cell proliferation, invasion, migration, translation, and other pathogenesis indicates their full potential as therapeutic targets. In addition, lncRNAs and circRNAs also interfere with the resistance of chemotherapy drugs in cancer cells, giving a novel strategy for cancer treatment. To date, lncRNAs have been studied more than circRNAs, and circRNAs will be a promising future trend. However, it is arduous to figure out lncRNAs and circRNAs functions and mechanisms. They can be preserved at a much lower rate than protein‐coding genes. And they can affect survival and regulate proliferation, apoptosis, differentiation, cell cycle, migration, invasion and metastasis, glycolysis, DNA methylation, and drug resistance. In addition, they can influence cancer risk and complex mechanisms, alter the chromosomal structure and regulate gene expression, chromatin modification, and remodeling. Recently, the most common method to identify lncRNAs and circRNAs is RNA‐seq. Though it is efficient, we still need to recognize the limitations, such as the differences in the results due to the various datasets, the need for more biological experiments, and clinical validations to further validate. With the advancement of biotechnology, other more efficient ways are looked forward to being used. Besides techniques, understanding the effects of various methods on the interpretation of lncRNA function is also necessary. At the same time, independent results between different researchers need to be validated.

Though there are quite a few studies on lncRNAs and circRNAs, their therapeutic potential is untapped. The differential expression characteristic enables them to be the biomarker in cancer. Moreover, the high stability feature in body fluid strengthens their feasibility in the treatment of nonintrusion detection. It is worth noting that research methods should not be used only as tools in the research field, but as therapeutics with clinical potential. Creating a clinical trial standard to verify the effective biomarker or therapeutic target can be a feasible means. Practically, the toxic effects, off‐target effects, the construction of a safe and efficient carrier all need to be taken into consideration. Future studies should focus on improving the delivery options of miRNAs and lncRNAs for prolonging therapeutic efficiency and safety. Besides viral gene delivery of siRNAs or overexpression of transcripts, antisense oligonucleotides can also be used to block lncRNA functions. Despite the presence of ASOs, the purpose of interfering with RNA–protein complexes is to therapeutically interfere with lncRNA function. Furthermore, using reliable preclinical animal models will probably help to accelerate the study of neuroblastoma therapy. With advances in the mechanism of action, oligonucleotide design, and targeting methods, it is promising to incorporate many therapeutic compounds targeting lncRNAs and circRNAs into the clinical treatment of cancer.

## CONFLICT OF INTEREST

The authors declare that they have no competing interests.

## AUTHOR CONTRIBUTIONS

X. Y. and H. L. collected the related literature and drafted the manuscript. L. M., Z. Z., and J. H. participated in the design of the review and drafted the manuscript. H. L., L. L., and Z. Z. revised and edited the manuscript. L. M., Z. Z., and J. H. supervised the review process. All authors have read and approved the final manuscript. X. Y. and H. L. contributed equally to this work.

## ETHICS STATEMENT

Not applicable.

## Data Availability

Not applicable.
